# A 10-km CMIP6 downscaled dataset of temperature and precipitation for historical and future Vietnam climate

**DOI:** 10.1038/s41597-023-02159-2

**Published:** 2023-05-06

**Authors:** Quan Tran-Anh, Thanh Ngo-Duc, Etienne Espagne, Long Trinh-Tuan

**Affiliations:** 1 Faculty of Environment, Hanoi University of Mining and Geology, Hanoi, Vietnam; 2 Department of Space and Applications, University of Science and Technology of Hanoi (USTH), Vietnam Academy of Science and Technology (VAST), Hanoi, Vietnam; 3 French Development Agency (AFD), Paris, France; 4 Center for Environmental Fluid Dynamics, VNU University of Science, Hanoi, Vietnam

**Keywords:** Projection and prediction, Natural hazards

## Abstract

High-resolution climate projections are mandatory for many applications and impact assessments in environmental and management studies. In response to the needs in Vietnam, this study constructs a new precipitation and temperature daily dataset for Vietnam, at a high spatial resolution of 0.1° × 0.1°, based on the outputs of 35 global climate models (GCMs) from the Coupled Model Intercomparison Project Phase 6 (CMIP6). The Bias Correction and Spatial Disaggregation (BCSD) method is adopted to bias-correct monthly GCM simulations using observation data, then subsequently temporally disaggregate them into daily data. The new dataset is called CMIP6-VN, covering the present-time period 1980–2014 and future projections for 2015–2099 from both CMIP6 tier-1 (Shared Socioeconomic Pathways (SSPs) 1–1.26, 2–4.5, 3–7.0, and 5–8.5) and tier-2 (SSPs 1–1.9, 4–3.4, 4–6.0) experiments. Results indicated the good performance of CMIP6-VN for the historical period, suggesting that the dataset could be used for studies on climate change assessment and impacts in Vietnam.

## Background & Summary

The Coupled Model Intercomparison Project (CMIP), established in 1995, is an effort of the World Climate Research Programme (WCRP) that produces and studies the outputs of Global Climate Models (GCMs) to better understand the past, present, and future of the climate system. The new state-of-the-art climate projections for the coming decades, made available by the CMIP Phase 6 (CMIP6)^1^, provide the underlying scientific ground for the latest IPCC Sixth Assessment Report (AR6)^2^ of the Intergovernmental Panel on Climate Change (IPCC). The Shared Socioeconomic Pathways (SSPs) scenarios used in AR6 include the SSP1 (Sustainability), SSP2 (Middle of the road), SSP3 (Regional Rivalry), SSP4 (Inequality), and SSP5 (Fossil-fueled Development) pathways^3^. The CMIP6 future scenario experiments are classified into core priority groups, including 1) the tier-1 experiments with SSPs 1–2.6, 2–4.5, 3–7.0, and 5–8.5, and the tier-2 experiments with SSPs 1–1.9, 4-3.4, 4–6.0, and 5–3.4^4^.

To date, the resolution of the CMIP6 GCMs is still too coarse (usually over 100 km) to be used in many aspects such as risk assessment, adaptation management, and decision-making procedures at the regional or local scale. Besides the coarse resolution, biases and uncertainties contained in GCMs often exaggerate from global to regional and local scales, restraining the usefulness and applicability of GCMs in small-scale studies^5–7^. Hence, downscaling techniques, which transform the coarse GCM information into a higher spatial resolution, should be conducted for a specific region of interest before conducting local impact assessment and risk management. There are two popular downscaling techniques, namely dynamical and statistical, that have been widely used. Dynamical downscaling is related to a modeling process that feeds the coarse-resolution initial conditions (IC) and lateral boundary conditions (LBC) provided by a GCM into a regional climate model (RCM) to produce higher-resolution climate information^8,9^. Along with the massive requirement of computer resources and modeling time, the dynamical downscaling method still has to deal with biases and the sensitivity of the boundary conditions taken from the host GCM, as well as the accuracy and uncertainty of the dynamics and physical parameterization of each RCM^8^. On the other hand, statistical downscaling amounts to firstly searching for a relationship between local observed variables (called predictands) and large-scale GCM climate predictors. Then by assuming that the derived relationship is maintained with time, we can feed predictors of GCM outputs into the statistical model to obtain future climate information^10,11^. The statistical downscaling approach is effective and generally does not require huge computer resources. To date, different statistical downscaling techniques have been widely adopted in various studies, e.g. climate risks and water resources, at local and regional scales^12–14^.

In Vietnam, the Ministry of Natural Resources and Environment (MONRE) has published several reports on Climate Change and Sea-level rise scenarios for Vietnam^15–18^. Constrained by computing resources, the results of the latest MONRE reports^15,16^ were only based on a limited number of dynamically downscaled experiments (14 experiments in total with 4 RCMs and 10 CMIP5 GCMs) and for only two Representative Concentration Pathways (RCP) scenarios RCP4.5 and RCP8.5. Besides, a statistical downscaling effort has been implemented in Vietnam to downscale 31 CMIP5 GCMs under four RCPs scenarios^19,20^.

In the present study, we produce a new high-resolution (10-km) climate dataset for Vietnam by statistically downscaling 35 CMIP6 GCMs and eight SSPs scenarios. The Biases Corrected Spatial Disaggregation (BCSD) method^21,22^ was applied. It is noteworthy that we were able to collect daily temperature and rainfall observed station data in order to generate a gridded-based observation dataset over Vietnam, which served as important and mandatory inputs for the BCSD scheme. The new dataset, called CMIP6-VN, contains downscaled values of 4 variables, i.e. daily rainfall (*pr*), daily mean 2m-temperature (*tas*), daily maximum 2m-temperature (*tasmax*), and daily minimum 2m-temperature (*tasmin*) for the historical period 1980–2014 and the future period 2015–2099. CMIP6-VN is expected to be a valuable input with the most updated information for studies on climate change assessment and impacts in Vietnam.

## Methods

### Study area

**Fig. 1:**
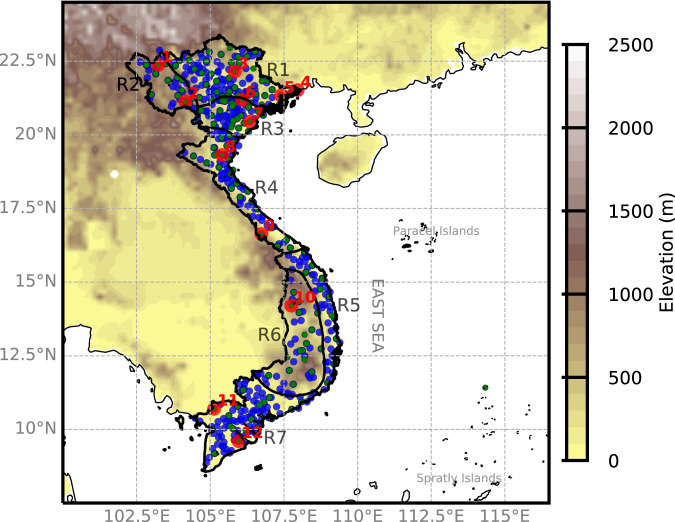
The study domain Vietnam and its 7 climate regions. Green and blue dots show the locations of 157 temperature and 481 precipitation stations, of which the data are used in this study. The red circles indicate 12 random stations selected for the validation of the OBS gridded dataset. The topography over Vietnam (shaded, in m) is obtained from the 5-minute Gridded Global Relief Data (ETOPO5)^46^.

We focus on Vietnam in this study. Vietnam has a diverse topography spanning over a long range of latitudes, a long coastal line, and a monsoon-influenced climate. Based on specific criteria for radiation, temperature, and rainfall^23,24^, Vietnam is divided into seven climatic regions, including (1) the Northwest (denoted as R1), (2) the Northeast (denoted as R2), (3) the Red River Delta (denoted as R3), (4) the North Central (denoted as R4), (5) the South Central (denoted as R5), (6) the Central Highland (denoted as R6), and (7) the South region (denoted as R7) (Fig. 1). In this study, the CMIP6-VN dataset is developed for the inland territory of Vietnam.

### Data acquisition

To guide the statistical downscaling process, we collected daily-observed precipitation (*pr*) and near-surface temperatures (daily average *tas*, daily maximum *tasmax*, and daily minimum *tasmin*) from 481 and 147 stations, respectively, of the Vietnam Meteorological and Hydrological Administration (VMHA) for the period 1980–2014 (Fig. 1). The data underwent prior verification by VMHA’s Documentation Center, following established operational processes. We subsequently applied the three-sigma (five-sigma) rule to identify any suspect values in the temperature (rainfall) data and re-examined each identified case.

Monthly rainfall and near-surface temperatures from 35 CMIP6 GCMs (Table 1) are downscaled for the historical period 1980–2014 and future period 2015–2099 under the eight SSPs 1–1.9, 1–2.6, 2–2.5, 3–4.0, 3–7.0, 4-3.4, 4-6.0, 5-3.4, and 5–8.5. The CMIP6 GCMs data are acquired via the Earth System Grid Federation website (ESGF, https://esgf-node.llnl.gov/projects/cmip6/).

**Table 1:** List of 35 CMIP6 GCMs and associated scenarios considered in this study.

No	Model name	Resolution (lat. × lon.)	Variant label	Historical	SSP1–1.9	SSP1–2.6	SSP2–4.5	SSP3–7.0	SSP4-3.4	SSP4–6.0	SSP5–8.5
1	ACCESS-CM2	1.88° × 1.25°	r1i1p1f1	x	-	x	x	x	-	-	x
2	ACCESS-ESM1-5	1.88° × 1.25°	r1i1p1f1	x	-	x	x	x	-	-	x
3	AWI-CM-1-1-MR	0.93° × 0.94°	r1i1p1f1	x	-	x	x	x	-	-	x
4	BCC-CSM2-MR	1.13° × 1.13°	r1i1p1f1	x	-	x	x	x	-	-	x
5	CAMS-CSM1-0	1.13° × 1.12°	r1i1p1f1	o	-	-	-	-	-	-	-
6	CAS-ESM2-0	2.81° × 2.81°	r1i1p1f1	o	-	o	-	o	-	-	o
7	CESM2	1.41° × 1.42°	r1i1p1f1	o	-	o	o	o	-	-	o
8	CESM2-WACCM	1.25° × 0.94°	r4i1p1f1	o	-	o	-	o	-	-	o
9	CIESM	1.25° × 0.94°	r1i1p1f1	x	-	x	x	-	-	-	x
10	CMCC-ESM2	1.25° × 1.25°	r1i1p1f1	x	-	x	x	x	-	-	x
11	CNRM-CM6-1-HR	1.25° × 0.94°	r1i1p1f1	x	-	x	x	x	-	-	x
12	CNRM-ESM2-1	0.5° × 0.5°	r1i1p1f2	x	x	x	x	x	x	x	x
13	CanESM5	1.41° × 1.39°	r1i1p1f2	x	x	x	x	x	x	x	x
14	EC-Earth3	0.7° × 0.7°	r1i1p1f1	x	-	x	x	x	-	-	x
15	EC-Earth3-Veg	0.7° × 0.7°	r1i1p1f1	x	x	x	x	x	-	-	x
16	FGOALS-f3-L	1.25° × 0.8°	r1i1p1f1	o	-	o	-	o	-	-	o
17	FGOALS-g3	2° × 2.03°	r1i1p1f1	x	x	x	x	x	x	x	x
18	FIO-ESM-2-0	1.25° × 1.25°	r1i1p1f1	x	-	o	-	-	-	-	o
19	GFDL-ESM4	1° × 1°	r1i1p1f1	x	x	x	x	x	-	-	x
20	GISS-E2-1-G	2.5° × 2.5°	r1i1p1f2	x	x	x	x	x	x	x	x
21	HadGEM3-GC31-LL	1.88° × 1.88°	r1i1p1f3	x	-	x	x	-	-	-	x
22	HadGEM3-GC31-MM	0.83° × 0.56°	r1i1p1f3	x	-	x	-	-	-	-	x
23	IITM-ESM	1.88° × 1.89°	r1i1p1f1	o	-	o	-	o	-	-	o
24	INM-CM5-0	2° × 1.5°	r1i1p1f1	x	-	x	x	x	-	-	x
25	IPSL-CM6A-LR	2.5° × 1.27°	r1i1p1f1	x	x	x	x	x	x	x	x
26	KACE-1-0-G	1.88° × 1.88°	r1i1p1f1	o	-	o	-	o	-	-	o
27	MCM-UA-1-0	3.75° × 2.24°	r1i1p1f2	o	-	o	-	o	-	-	o
28	MIROC-ES2L	1.41° × 1.41°	r1i1p1f1	x	x	x	x	x	-	-	x
29	MIROC6	2.81° × 2.77°	r1i1p1f2	x	x	x	x	x	x	x	x
30	MPI-ESM1-2-HR	0.94° × 0.94°	r1i1p1f1	x	-	x	x	-	-	-	x
31	MRI-ESM2-0	1.13° × 1.13°	r1i1p1f1	x	x	x	x	x	x	x	x
32	NESM3	1.88° × 1.88°	r1i1p1f1	x	-	x	x	-	-	-	x
33	NorESM2-MM	1.25° × 0.94°	r1i1p1f1	o	-	o	o	o	-	-	o
34	TaiESM1	1.25° × 0.94°	r1i1p1f1	o	-	-	-	o	-	-	o
35	UKESM1-0-LL	1.88° × 1.25°	r1i1p1f2	x	x	x	x	x	-	-	x

Note:

x: available for both precipitation and temperature

o: only precipitation

-: not available

### Building the gridded observation dataset

We interpolated the observed station data of rainfall and temperature into a 0.1° × 0.1° gridded dataset (hereafter called OBS) using the Spheremap^25^ and Kriging^26^ interpolation techniques, respectively. For rainfall, the Spheremap method has some advantages over other interpolation techniques such as Cressman^27^, Inverse Distance Weighted^28^, or Kriging^26^. Similarly, the Kriging method is more suitable for interpolating continuous spatial variables such as temperature^29^. The OBS dataset is subsequently used to bias-correct the GCM CMIP6 data.

### Downscaling process

In this study, the BCSD method^21,22^ is applied for downscaling the CMIP6 GCM outputs for Vietnam. The BCSD consists of two major steps: bias correction (BC) and spatial disaggregation (SD), which are briefly described below.

Before the BC step, all GCM data and the OBS are regridded to the intermediate resolution of 1° × 1°. We detrend the temperature data at each grid point before the BC, then add the trends back afterward to preserve the climatic trends in the original GCM^21,30,31^.

The BC firstly applies the quantile mapping (QM) method^32^, which corrects the biases in the GCM data when compared to the OBS at the resolution of 1° × 1°. For each variable on each grid cell in each month, cumulative distribution functions (CDFs) for both the OBS and historical GCM data are separately generated. Transfer functions (TFs) that map the model CDFs onto the OBS CDFs are subsequently developed. Then, the biases in the GCM monthly outputs are corrected by applying the TFs to transform the GCM data to the corresponding OBS data of the same CDF quantile. Those QM TFs are assumed to be stable through the historical and future periods and thus are applied to correct the future projected variables. For temperatures, after the QM step, the previously saved climatic trends are added back to the QM model data. Then, the climatological mean bias adjustment between the GCM and the OBS temperature at each grid point is subsequently applied, producing the BC temperature data at the intermediate resolution of 1° × 1°.

In the SD step, the BC data are interpolated to the resolution of 0.1° × 0.1° following a three-step procedure: (1) bilinearly interpolating the additive (for temperatures) and multiplicative (for precipitation) change factors estimated between the BC GCM fields and the OBS climatology to the targeted high-resolution of 0.1° × 0.1°; (2) constructing the high-resolution BC GCM data by adding (for *tas*, *tasmax*, and *tasmin*) or multiplying (for *pr*) the interpolated change factors and the 0.1° × 0.1° OBS climatology; (3) finally, the monthly BC fields of the future period are temporally disaggregated to a daily scale by randomly choosing a respective month from OBS and additively (for temperatures) and multiplicatively (for precipitation) adjusting its daily values to reproduce the future monthly BC data. Note that for precipitation downscaling, the temporal disaggregation requires an assessment of the scaling factor and the number of wet days to avoid unrealistic precipitation values. For example, if the number of wet days in the selected month is less than three and its scaling factor is greater than three, another year with more wet days in that month will be selected.

The implementation of the BCSD approach in this study contains two main phases:

- Phase 1 — Testing: the climatological fields of the training period 1980–2004 from the OBS and GCMs are used to develop the TFs between the simulations and observations. Then, the BCSD is applied to the independent (testing) period 2005–2014, and the results (hereafter called BCSD-CMIP6) are compared with OBS to examine the performance of the BCSD in reproducing past climate conditions.

- Phase 2 — Future downscaling: To maximize the construction period of the BCSD approach^33,34^, the total 35 years from 1980 to 2014 are used to generate the TFs and to guide spatial disaggregation for the future period 2015–2099. The BCSD is applied to all GCM models and SSPs scenarios listed in Table 1 to generate the targeted CMIP6-VN dataset.

## Data Records

The generated OBS and CMIP6-VN datasets are stored in the figshare repository^35^. Both datasets include daily values of rainfall (*pr*) and temperatures (*tas*, *tasmax*, and *tasmin*) and cover the historical period of 1980–2014. In addition, CMIP6-VN provides future projections for 2015–2099 with the 7 SSPs. The datasets are in the Network Common Data Form (netCDF) classic format. For CMIP6-VN, one compressed tar file is created for each model, in which each variable for each historical/scenario period is saved in one netCDF file. The total data size for OBS and CMIP6-VN is 480 Mb and 76.31 Gb, respectively.

## Technical Validation

### The OBS gridded dataset

**Fig. 2:**
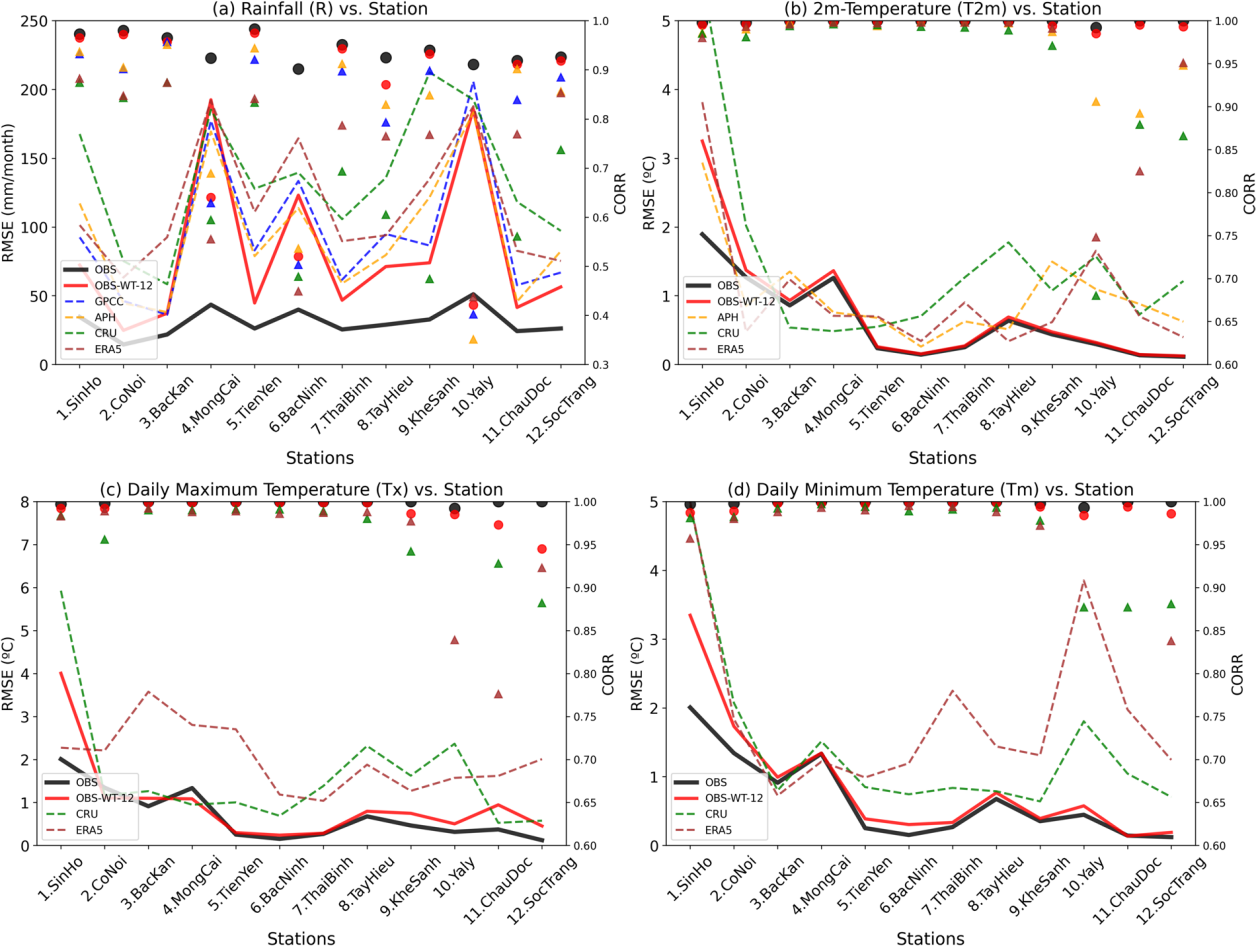
RMSE (mm d^−1^, left axis), and correlation (right axis) at the 12 randomly selected stations between the gridded datasets and the gauge observation.

The OBS gridded dataset was constructed utilizing daily rainfall and temperature data from 481 and 147 stations, respectively. In order to validate the quality of the OBS data, we employed the methodology presented in Nguyen-Xuan *et al*.^36^ by randomly selecting 12 stations in the sub-climatological regions in Vietnam (see Fig. 1 and Supplementary Figure S1). These 12 stations were removed from the set of stations used to interpolate the OBS gridded dataset; and with the remaining set of stations, we applied the same interpolation algorithm to create a testing dataset, which we refer to as OBS-WT-12. Monthly rainfall and temperature data from OBS and OBS-WT-12 at these 12 stations during the period of 1980–2014 were compared with the gridded datasets typically used in the study region (Fig. 2). These datasets include: (1) the monthly Global Precipitation Climatology Centre (GPCC)^37^, (2) the daily Asian Precipitation – Highly-Resolved Observational Data Integration Towards Evaluation (APHRODITE)^38^, (3) the monthly Climate Research Unit (CRU) data^39^, and (4) the daily ERA5-Land data (hereafter called ERA5)^40^, which is the fifth generation European Centre for Medium-Range Weather Forecasts (ECMWF) atmospheric reanalysis. Noting that GPCC only provides rainfall data, while only CRU and ERA5 provide data on daily maximum and minimum temperatures. The horizontal resolution of OBS, OBS-WT-12, and ERA5 is 0.1°, while APHRODITE has a resolution of 0.25°, and GPCC and CRU have a resolution of 0.5°.

Figure 2 illustrates the superior performance of OBS over the other datasets based on statistical parameters, namely the root mean square error (RMSE) and correlation (CORR), when compared to *in-situ* data from the 12 selected stations. Even without the use of inputs from these 12 stations, OBS-WT-12 outperforms the remaining datasets. For temperature, the correlations of OBS and OBS-WT-12 with *in-situ* data are approximately equal to 1, while for rainfall, they are greater than 0.9 across most stations. Meanwhile, the correlation of the remaining datasets is lower, particularly for rainfall across all stations and for temperature at stations numbered 9–12 (Table 2), located in the central and southern parts of Vietnam.

**Table 2:** Locations of 12 stations randomly selected for validating the OBS dataset.

#	Station name	Lon (°E)	Lat (°N)	Height (m)
1	SinHo	103.23	22.37	1529
2	CoNoi	104.15	21.13	704
3	BacKan	105.83	22.15	174
4	MongCai	107.97	21.52	7
5	TienYen	107.40	21.33	14
6	BacNinh	106.08	21.18	8
7	ThaiBinh	106.35	20.45	3
8	TayHieu	105.40	19.32	72
9	KheSanh	106.73	16.63	367
10	Yaly	107.75	14.20	547
11	ChauDoc	105.13	10.70	5
12	SocTrang	105.97	9.60	3

The value of RMSE varies across stations, with OBS performing the best. For rainfall, OBS-WT-12 has a smaller RMSE than the other remaining datasets, except for the MongCai station where the OBS_WT-12 RMSE is slightly higher. For temperature, the OBS and OBS-WT-12 RMSE outperform the other datasets. At the SinHo station, the RMSE value is much higher than other stations, possibly due to its high altitude of 1529 m, leading to the fact that the observed temperature at SinHo does not represent the temperature of the entire grid. Noting that we have adjusted the temperature value with its topography dependence when applying the Kriging interpolation method by using an environmental lapse rate of 0.65 °C/km.

In brief, the results from Fig. 2 demonstrate that OBS performs well not only in grids with monitoring stations but also significantly better than the commonly used gridded datasets in the study region over the grids where there are no stations for rainfall and temperature.

### The CMIP6-VN dataset

#### Spatial distribution

**Fig. 3:**
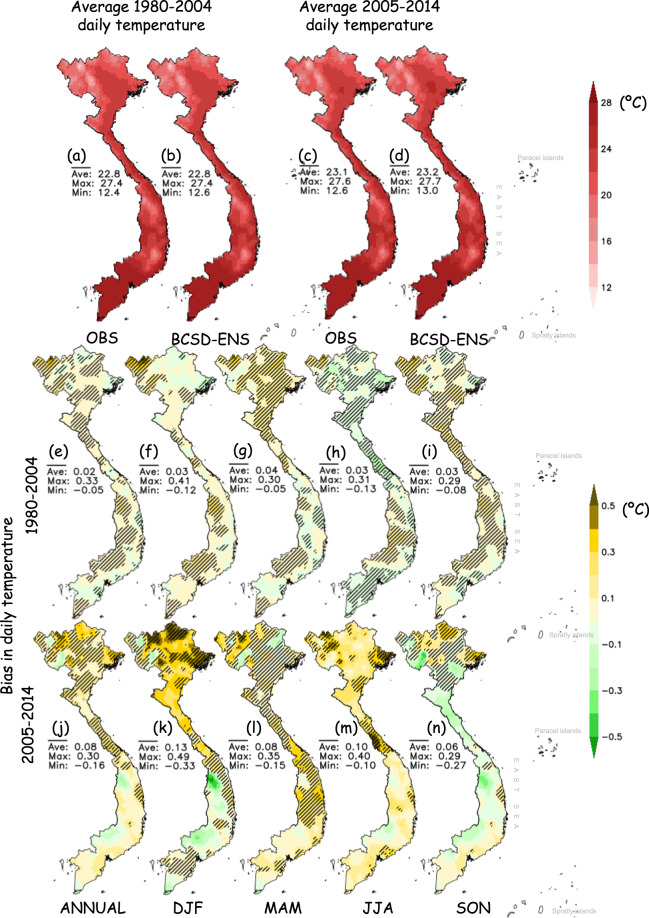
Spatial distribution of the average temperature in Viet Nam; (**a,** **b**) and (**c,** **d**) indicate the average temperature of 1980–2004 and 2005–2014 by OBS and the BCSD-ENS, respectively; (**e**–**i**) and (**j**–**n**) show the biases of the BCSD-ENS compared to OBS for the training period 1980–2004 and the testing period 2005–2014. Hatched lines show the regions in which more than two-thirds of the CMIP6 models have the same sign as the BCSD-ENS. Statistical values (average, maximum, minimum) over the entire Vietnam inland territory are also displayed.

Figure 3 displays the spatial distribution of observed and modeled 2m-temperature and their difference over Vietnam. The modeled 2m-temperature is derived from the ensemble mean of the 35 BCSD downscaled CMIP6 GCMs described in Table 1 (hereinafter referred to as BCSD-ENS). There are high similarities between the BCSD-ENS and OBS in both training and testing periods. The model ensemble effectively reproduces the slight temperature shift between regions where the temperature tends to increase from North to South and reaches the highest value in Southern Vietnam. The annual and seasonal biases, given by the difference between the BCSD-ENS and OBS, range from −0.26 °C to 0.82 °C and from −0.67 °C to 0.98 °C for the training (Fig. 3e–i) and testing periods (Fig. 3j–n), respectively. The overall average annual bias is negligible, reaching only 0.05 °C (0.16 °C) for the training (testing) period. It is worth noting that the seasonal temperature biases are more pronounced than the annual average biases (middle and lower panels of Fig. 3), and the biases in the testing period are more pronounced than those in the training period. The seasonal temperature biases are larger in the northern regions than in the southern regions, especially in the testing period. It should be noted that the bias values shown in Fig. 3 are very small compared to those of non-bias-corrected and higher resolution simulations over the region^41–44^. For example, the absolute temperature biases of an ensemble of dynamical downscaling simulations^42^ are generally between 0.5–1 °C, which are much larger than the biases obtained with the BCSD products in this study. In brief, although biases are unavoidable in the BCSD products, the overall average annual and seasonal temperature biases over Vietnam are relatively small, suggesting the appropriate quality of the BCSD temperatures.

The biases of the BCSD-ENS daily maximum and minimum temperatures are provided in the online supplemental materials (Supplementary Figures S1, S2). The biases of these fields, although generally higher than those of 2m-temperature in both annual and seasonal averages, are still small enough to demonstrate the good performance of the BCSD products.

**Fig. 4:**
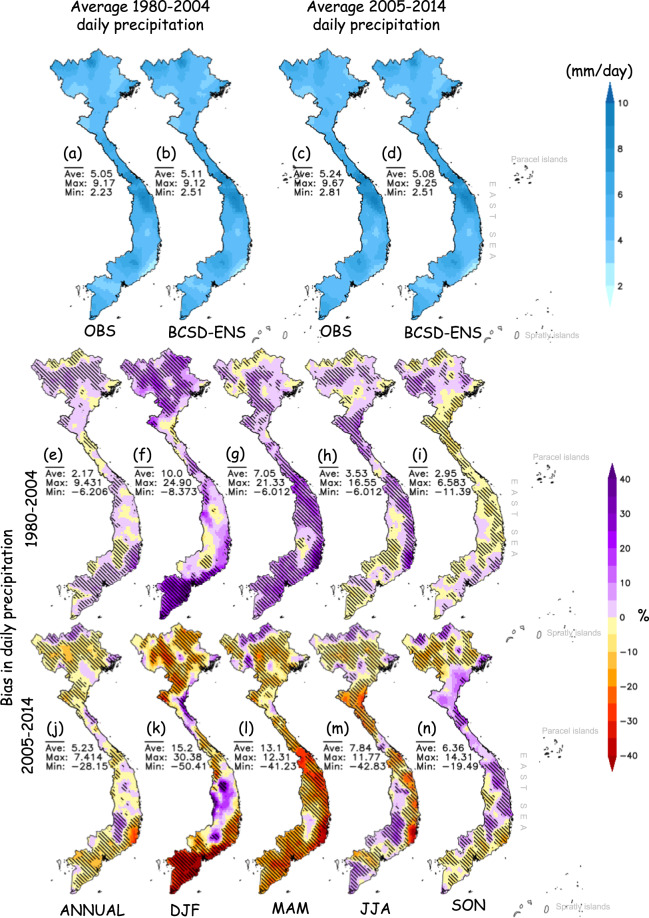
Same as Fig. 3 but for precipitation.

Simulated precipitation climatology by the BCSD-ENS well agrees with OBS, which can be seen via the relatively low annual bias of 2.17% and 5.23% in the training (Fig. 4e) and testing (Fig. 4j) periods, respectively. Locations of dry regions (i.e. the Northeast and South of the South Central) and the wet regions (i.e. North of the Northeast and Central) are accurately captured by the BCSD outputs (Fig. 4a–d). The simulation biases are non-uniformly distributed between seasons and regions, and generally larger in the testing period than in the training period. The BCSD-ENS precipitation for the dry season of December-January-February (DJF) exhibits the largest bias compared to the other seasons. The DJF biases could reach 30.38% in the Central Highlands and-50.41% in the South during the testing period (Fig. 4k), and 24.9% in the north and −8.37% in the Central Highlands during the training period (Fig. 4f). However, it should be noted that a large relative bias (in %) could result from a small absolute bias value (in mm), especially in a dry region where a little change in precipitation often results in a significant relative bias.

#### Seasonal cycles

**Fig. 5:**
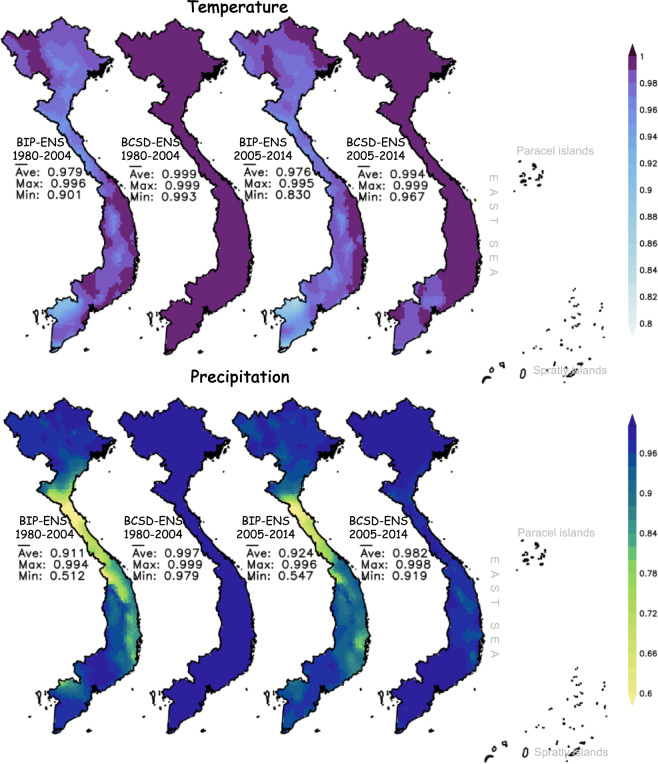
Temporal correlations of the temperature (upper) and precipitation (lower) seasonal cycles between the BCSD-ENS and BIP-ENS with OBS for the training period 1980–2004 (two left figures) and the testing period 2005–2014 (two right figures).

The reproducibility of the BCSD-ENS for seasonal cycles is examined by calculating the temporal correlations between the simulation and observation (Fig. 5). The average correlation values for the testing period are very high, over 0.994 for temperature and 0.982 for precipitation, indicating the good performance of the downscaled products (Fig. 5d,h). The overall performance of the BCSD-ENS is slightly better in the training period than in the testing period. The BCSD-ENS is further compared to the simple bilinear interpolation products (BIP), bilinearly interpolated from the CMIP6 GCM outputs onto the grid of 0.1° × 0.1° over Vietnam. The ensemble average of the BIP products (BIP-ENS) also shows a good agreement with OBS, which is partly illustrated by the high correlation coefficient of 0.976 and 0.926 for the seasonal cycles of temperature and precipitation, respectively, in the testing period (Fig. 5c,g). The BCSD-ENS generally exhibits better performance compared to the BIP-ENS. The skill of the BCSD-ENS in reproducing the seasonal cycle is also better for temperature than precipitation.

**Fig. 6:**
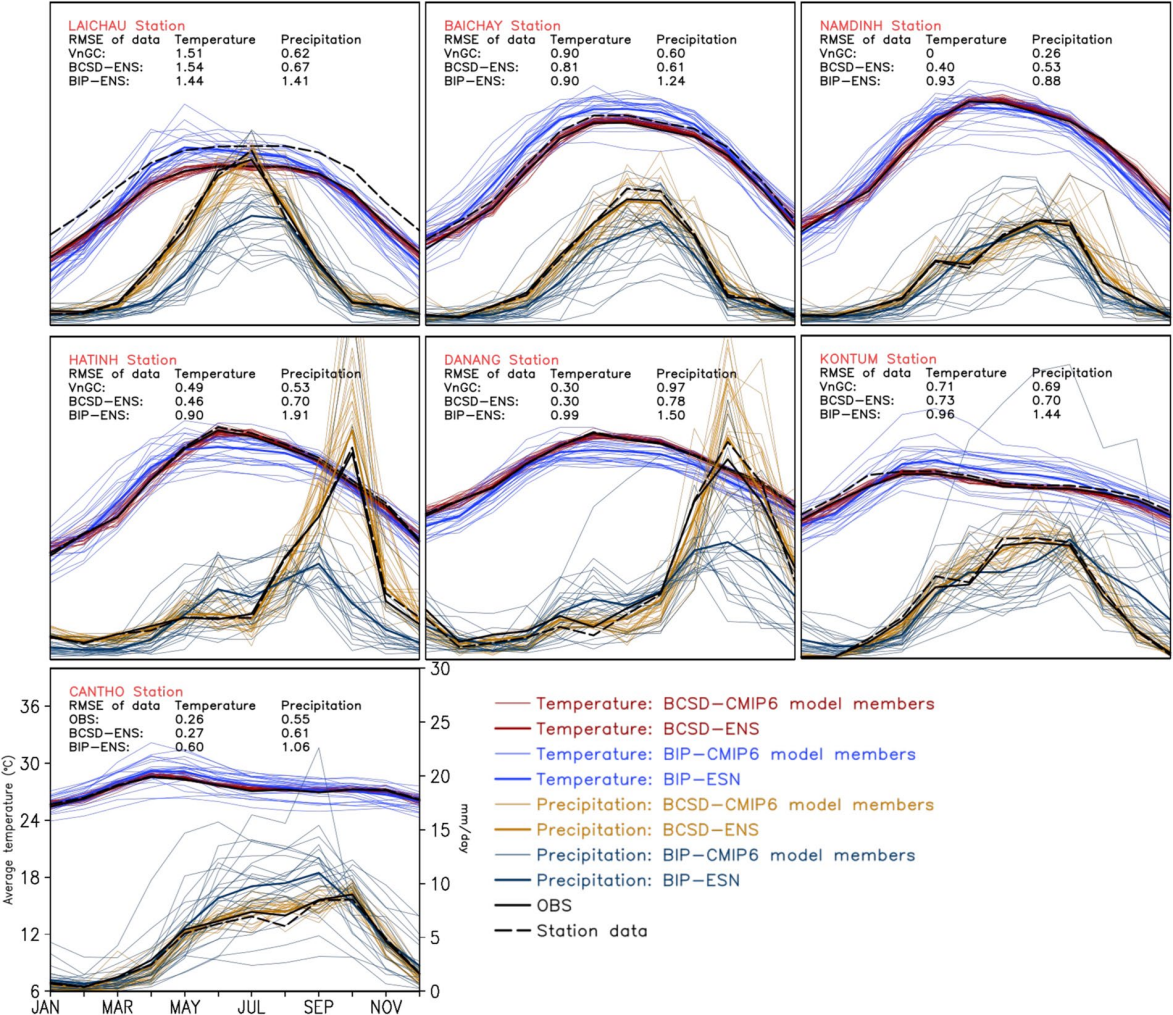
Comparison of the seasonal temperature/precipitation cycles according to station data, OBS, BCSD outputs, and their ensemble mean, and BIP outputs and their ensemble mean for seven station locations in the period 2005–2014. Values of mean square errors (MSE) of the BCSD-ENS, BIP-ENS, and OBS are also displayed.

We compare the BCSD-ENS, the BIP-ENS, the downscaled individual models by both BCSD and BIP methods, and OBS to examine further the ability of the downscaled product in reproducing the seasonal temperature cycles of the testing period (Fig. 6). The comparison was conducted at seven stations randomly taken from the list of stations located in the seven climatic sub-regions, including Lai Chau (Northwest), Bai Chay (Northeast), Nam Dinh (Red River Delta), Ha Tinh (North Central), Da Nang (Central), Kon Tum (Central Highlands) and Can Tho (South). There are good agreements between OBS and the observed station data, illustrated by low root mean square error (RMSE) values ranging from 0.26 °C in Can Tho to 1.51 °C in Lai Chau. The RMSE values between the BCSD-ENS and OBS are small, e.g. only 0.3 °C in Da Nang, indicating good agreement between OBS and the BCSD outputs. On the other hand, the BIP-ENS generally overestimates spring-summer temperature in the northern part of Vietnam and consistently underestimates autumn-winter temperature in the remaining months and regions. The RMSEs of the BIP-ENS, ranging from 0.6 °C in Da Nang to 1.44 °C in Lai Chau, are larger than those of the BCSD-ENS. Note that the dispersions among the BIP members are also larger than those among the BCSD members,

Regarding precipitation, the BCSD outputs can effectively capture the temporal variation and rainfall amount in all stations, including Ha Tinh (North Central) and Da Nang (Central), where the rainy season comes between two to three months later than the other regions of the country (Fig. 6). In months with high rainfall amounts, the dispersions among the downscaled products are larger. The average RMSEs of the BCSD-ENS outputs, ranging from 0.53 mm per day (Nam Dinh) to 0.78 mm per day (Da Nang), are much smaller than those of the BIP outputs (0.88–1.91 mm per day). The BCSD outputs consistently outperform the BIP outputs in all regions and seasons.

#### Added values

To confirm the superior performance of the BCSD method compared to the traditional and effortless BIP approach, we utilized the added value (AV) metric^45^, as presented below:

1 $$AV={\left({X}_{BIP}-{X}_{OBS}\right)}^{2}-{\left({X}_{BCSD}-{X}_{OBS}\right)}^{2}$$

where *X* represents one of the four downscaled variables. A positive AV indicates that the BCSD method outperforms the BIP approach, and vice-versa.

Positive AV grids dominate the majority of Vietnam’s mainland across all BCSD model outputs (Supplementary Figures S3, S4). For temperature, the percentage of grids with positive AVs ranges from 88.9% (EC-Earth3) to 98.2% (INM-CM5-0). Regarding rainfall, the percentage of positive AV grids varies from 85.9% (MIROC6) to 98% (FGOALS-g3). In brief, the BCSD method outperforms the BIP method in almost all regions of Vietnam for each CMIP6 GCM, and for the ensemble mean.

### Future projections

**Fig. 7:**
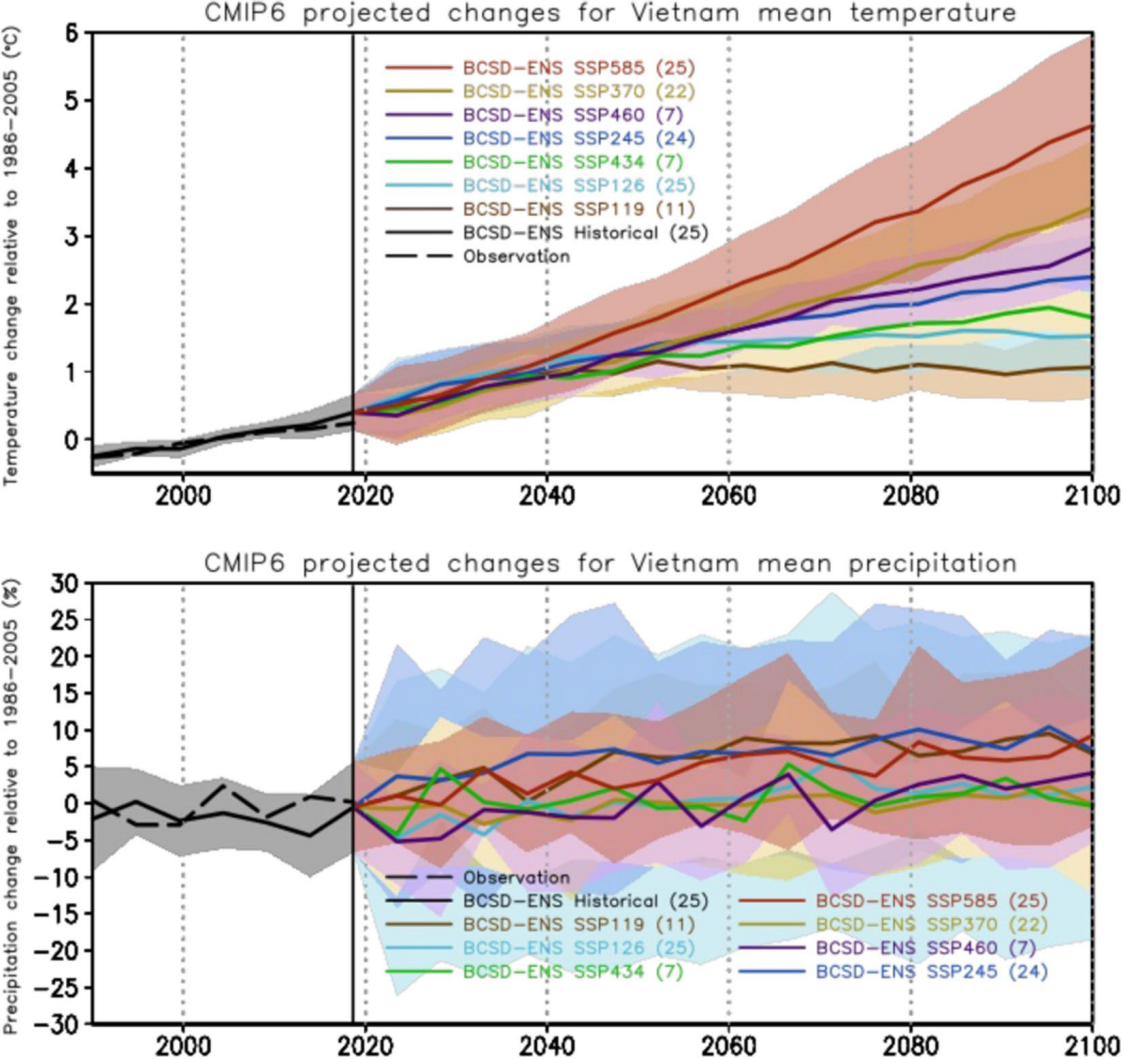
Projected changes relative to the baseline period 1986–2005 based on the CMIP6-VN data for temperature (upper) and precipitation (lower). Five-year moving averages are applied. Colored lines show the ensemble means of the models and colored shaded areas represent the areas of uncertainty (1 standard deviation) for each scenario. The number of models used for each scenario is given in bracket.

The range of uncertainty among the BCSD downscaled CMIP6 GCMs for the historical (1986–2014) and future period (2015–2099), illustrated by one standard deviation away from the mean, is displayed in Fig. 7. The averaged dispersion of the BCSD downscaled products in the historical period is relatively small, i.e. ±0.21 °C for temperature and ±5.1% for precipitation. The clear warming trend toward the end of the 21st century is projected by all SSPs, along with the growth of model uncertainty. Contrary to the clear increasing temperature trends across all models, the BCSD-ENS shows a slight increasing precipitation trend over entire Vietnam in the late 21st century with much larger uncertainties.

## Usage Notes

The sample scripts and the CMIP6-VN products are available for download. The GCM input variables (*pr*, *tas*, *tasmax*, and *tasmin*) and the gridded observation dataset should be prepared following the guidelines provided together with the script and dataset (please refer to the file readme.txt in the GitHub link). The step-by-step guide to the BCSD process is also described in detail in the readme.txt file. It should be noted that the sample script is prepared for downscaling the ACCESS-CM2 outputs for the 2015–2099 period under the SSP5-8.5 scenario. The script can be applied for any GCM and any historical/SSP scenario at any time frame with minor adjustments.

The CMIP6-VN dataset provides high resolution and multiple climate change scenarios, but it is important to acknowledge its limitations. Specifically, the QM step of the BCSD method may amplify the tails of the distribution in certain situations, potentially leading to under/overestimation of extreme events. Furthermore, the temporal disaggregation step may constrain future changes in the distribution of temperature or precipitation, such as changes in extreme event frequency or intensity. These limitations should be taken into account when interpreting results and utilizing the CMIP6-VN dataset for impact assessments.

## Supplementary information

Supplementary Information

## Data Availability

The code used to produce the CMIP6-VN dataset by downscaling the CMIP6 GCMs is publicly available at: https://github.com/quanta1985/Bias-Correct-and-Spatial-Dissaggregation. The code is written using Bash, R, and CDO scripts.
