# Supplementary material for: A 10-km CMIP6 downscaled dataset of temperature and precipitation for historical and future Vietnam climate
Source: Sci Data. 2023 May 6;10:257. doi: 10.1038/s41597-023-02159-2 (PMC10164165; doi:10.1038/s41597-023-02159-2)
Supplement: Supplementary file 1 — Supplementary Information [file 41597_2023_2159_MOESM1_ESM.pdf]

Supplementary Information

Figure S1. Spatial distribution of the average maximum daily temperature in Viet Nam; (a, b) and (c, d) indicate the average maximum daily temperature of 1980–2004 and 2005–2014 by OBS and the BCSD-ENS, respectively; (e–i) and (j–n) show the biases of the BCSD-ENS compared to OBS for the training period 1980–2004 and the testing period 2005–2014. Hatched lines show the regions in which more than two-thirds of the CMIP6 models have the same sign as the BCSD-ENS. Statistical values (average, maximum, minimum) over the entire Vietnam inland territory are also displayed. 2

Figure S2. Similar to Figure S1 but for minimum daily temperature. 3

Figure S3. AVs of 25 individual BCSD-CMIP6 models and BCSD-ENS to BIP results for temperature over the period 2005–2014. Positive (negative) AVs indicate better (worse) performance of the BCSD method. The percentage of grids with positive Avs is shown in the top right corner of each sub-figure. 4

Figure S4. Same with Figure S3 but for precipitation (35 models) 5

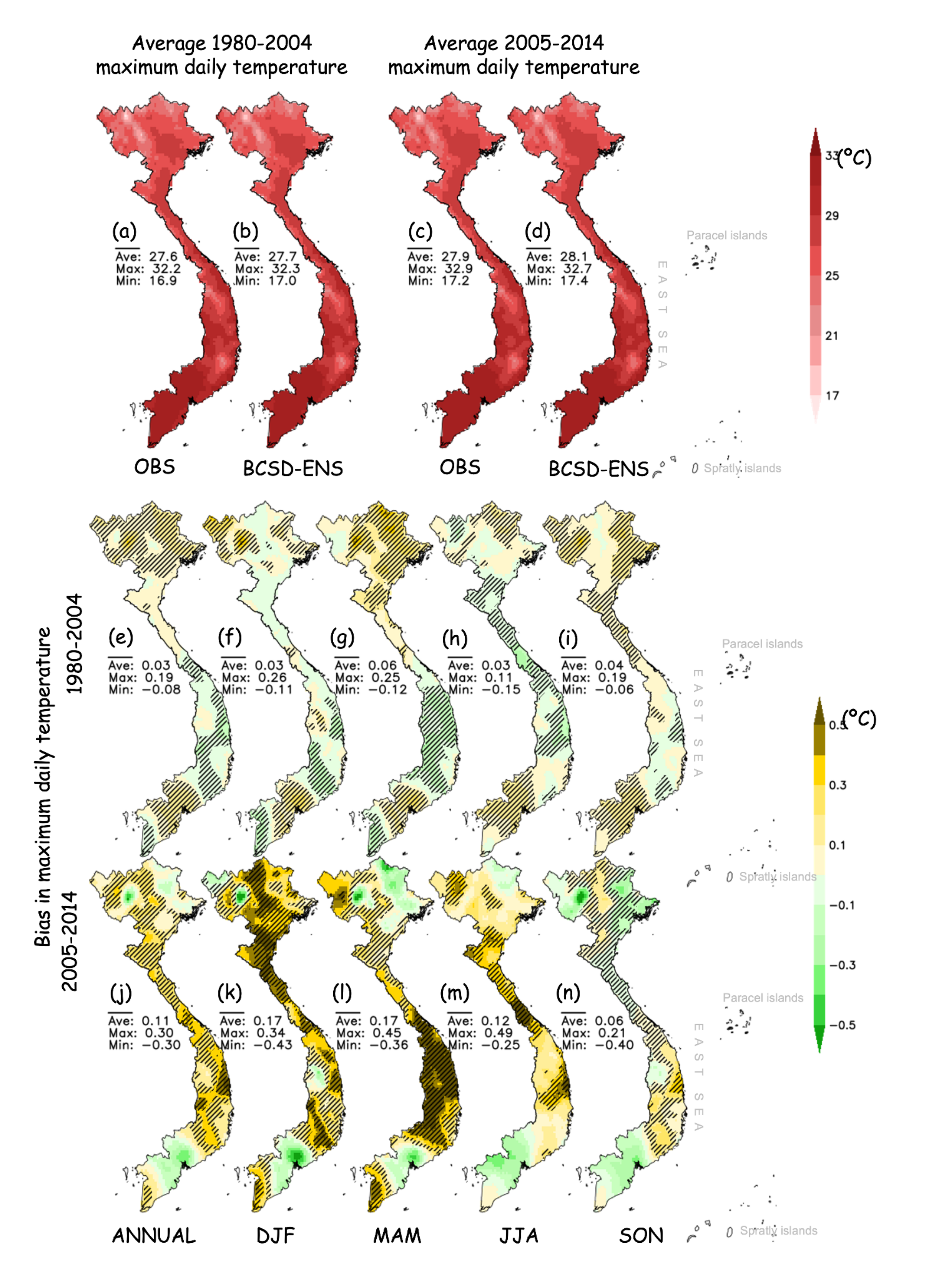

Figure S1. Spatial distribution of the average maximum daily temperature in Viet Nam; (a, b) and (c, d) indicate the average maximum daily temperature of 1980–2004 and 2005–2014 by OBS and the BCSD-ENS, respectively; (e–i) and (j–n) show the biases of the BCSD-ENS compared to OBS for the training period 1980–2004 and the testing period 2005–2014. Hatched lines show the regions in which more than two-thirds of the CMIP6 models have the same sign as the BCSD-ENS. Statistical values (average, maximum, minimum) over the entire Vietnam inland territory are also displayed.

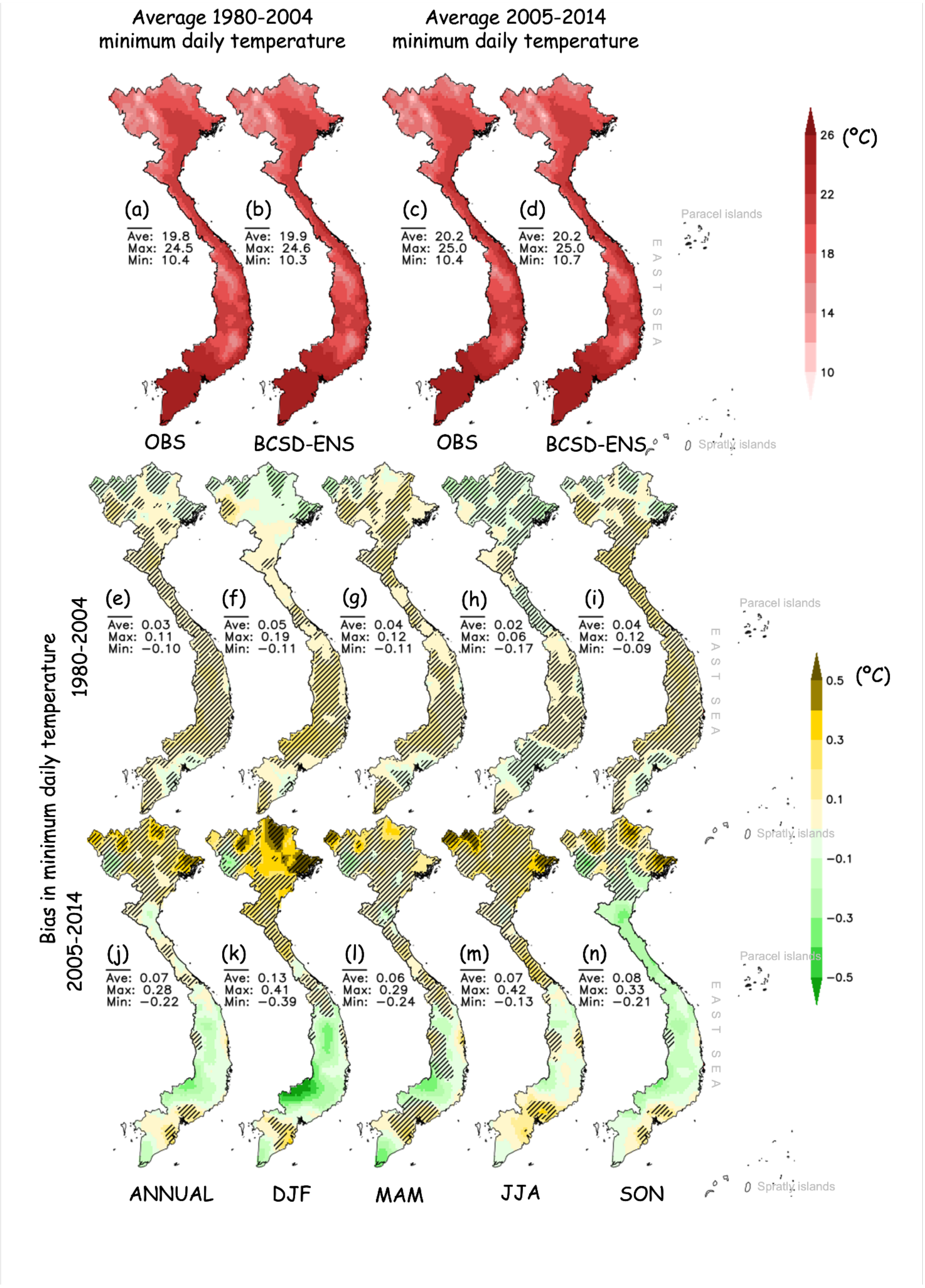

Figure S2. Similar to Figure S1 but for minimum daily temperature.

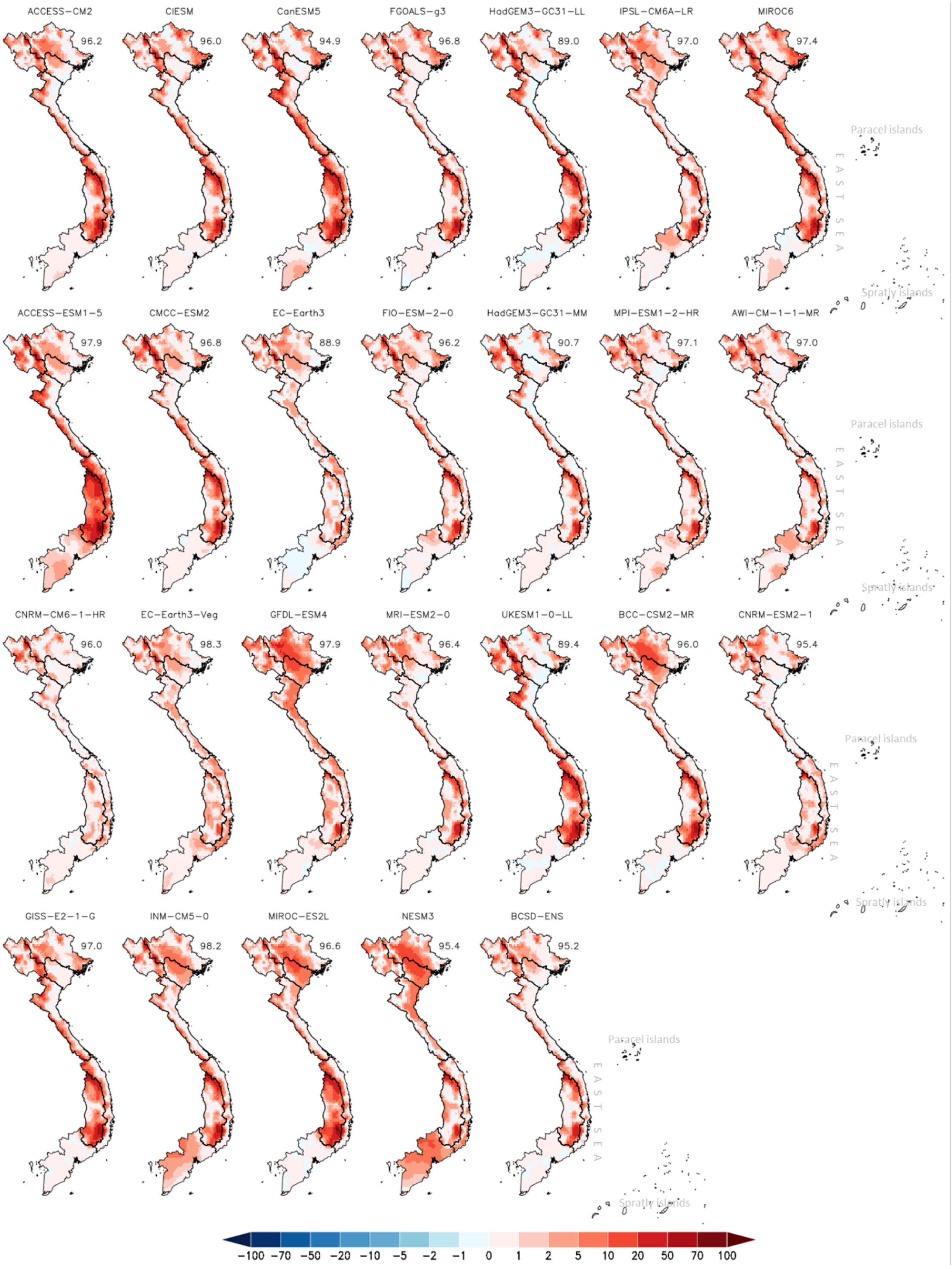

Figure S3. AVs of 25 individual BCSD-CMIP6 models and BCSD-ENS to BIP results for temperature over the period 2005–2014. Positive (negative) AVs indicate better (worse) performance of the BCSD method. The percentage of grids with positive Avs is shown in the top right corner of each sub-figure.

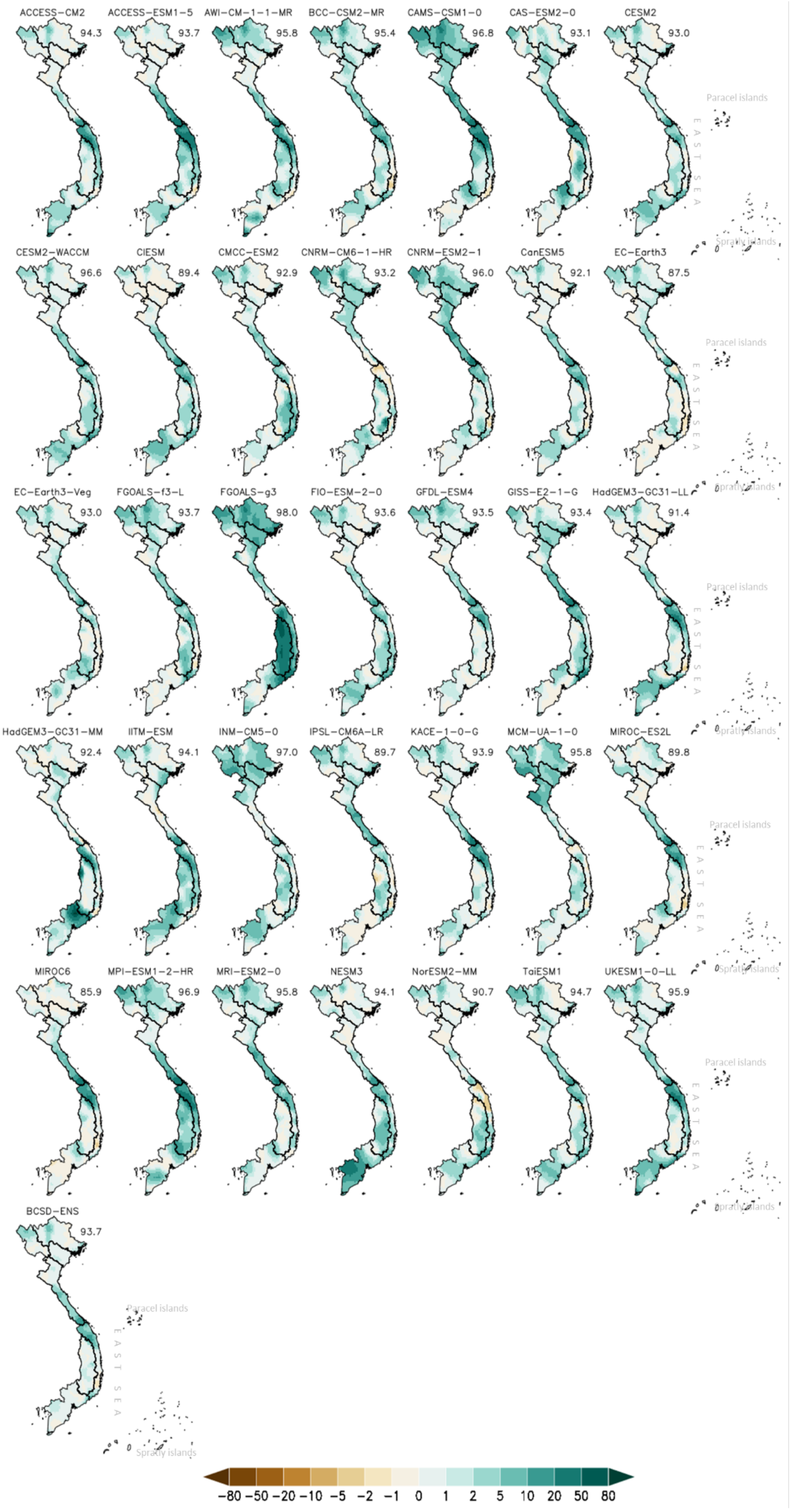

Figure S4. Same with Figure S3 but for precipitation (35 models)
